# Gender differences in the associations of recreational walking with indoor and outdoor falls among older adults-the Healthy Aging and Neighborhood Study (HANS)

**DOI:** 10.3934/publichealth.2025012

**Published:** 2025-02-08

**Authors:** Lingming Chen, Elizabeth Procter-Gray, Qun Le, Danielle LoPilato, Marianella Ferretto, Kevin Kane, Marian T. Hannan, Sarah Berry, Wenjun Li

**Affiliations:** 1 Department of Public Health and Center for Health Statistics, University of Massachusetts Lowell, 61 Wilder St, Lowell, MA 01854, USA; 2 Marcus Institute for Aging Research, Hebrew SeniorLife, 1200 Centre Street, Boston, MA 02131, USA

**Keywords:** recreational walking, indoor and outdoor falls, gender differences, fall prevention, healthy aging

## Abstract

**Objective:**

This study examined gender differences in the association between recreational walking and indoor and outdoor fall rates among older adults.

**Methods:**

The Healthy Aging and Neighborhood Study is a prospective cohort that included 716 community-dwelling adults aged 65–95 years in central and northeastern Massachusetts, USA (2018–2023). Recreational walking at baseline was measured by the frequency of walking for exercise for at least 10 min in the participants' neighborhood. Falls were reported on monthly falls calendars, and the circumstances for reported falls were collected via subsequent telephone interviews. Mixed effects negative binomial models were used to estimate gender differences in the associations of recreational walking with rates of indoor and outdoor falls, separately. Models were adjusted for sociodemographic variables, physical health, functional status, lifestyle behaviors, mental health, and fear of falling.

**Results:**

There were 394 (55%) female and 322 (45%) male participants enrolled in the study, and the *mean* (*SD*) age was 74.08 (6.29). About 61% of participants engaged in recreational walking at least once weekly. Women had lower outdoor fall rates than men (32 *vs*. 40 per 100 person-years), while indoor fall rates did not significantly differ by gender (31 *vs*. 34 per 100 person-years). Women engaging in recreational walking at least once weekly had a 62% lower indoor fall rate [IRR (95% *CI*): 0.38 (0.21, 0.71)] than those who did not. No significant associations were observed between recreational walking and outdoor falls for both women and men.

**Discussion:**

Among community-dwelling older women, but not men, a higher frequency of recreational walking was associated with lower rates of indoor falls, while no changes were seen with outdoor falls. Increasing recreational walking may be a viable focus for fall prevention programs in the community, especially for older women.

## 1. Introduction

Falls are a major health concern for older adults aged 65 years or older. In the United States, about one-third of older adults fall each year, with one-fifth reporting severe fall-related injuries such as fractures or head traumas [1],[2]. The medical cost of treating fall-related injuries is projected to increase from $35 in 2012 to $101 billion in 2030 [3]. Falls also affect older adults' functional capacities, reduce both mobility and quality of life, and even increase mortality rates [1],[4],[5]. In 2021, there were 38,742 deaths as a result of unintentional falls in older adults, and it is estimated that seven older adults may die from falls every hour by 2030 [6],[7]. Furthermore, the profiles for indoor and outdoor falls are quite different. Indoor falls occur more often among frail older adults, whereas outdoor falls are more frequent among active older adults [8],[9]. Only one study found that women reported lower outdoor fall rates but higher injurious indoor fall rates than men [10]. Walking is the most common type of physical activity and a recommended activity for community-dwelling older adults as it is convenient, cost-effective, and adaptable. Walking can serve two main purposes: utilitarian and recreation. Utilitarian walking refers to walking for essential errands or daily life tasks such as going to the grocery store, post office, or bank. Recreational walking refers to walking for exercise or leisure [11]. Adults do less utilitarian walking but more recreational walking as they age [12]. Gender differences in recreational walking are complex; some studies found that more older men did recreational walking than women, but others did not find any differences [13],[14].

However, the association between recreational walking and indoor and outdoor fall rates has not been well-studied, and the possible gender differences in these relationships remain unknown. Using data from the Healthy Aging and Neighborhood Study (HANS) prospective cohort study, we estimated gender differences in the association between recreational walking and indoor and outdoor falls among community-dwelling older adults living in Massachusetts, USA. The three hypotheses are as follows: a) Hypothesis a: higher frequency of recreational walking is associated with lower rates of indoor and outdoor fall; b) Hypothesis b: The association between recreational walking and indoor fall rate is stronger in women than men; c) Hypothesis c: The association between recreational walking and outdoor fall rate is stronger in men than women.

## 2. Materials and methods

### 2.1. Study design and participants

HANS is a longitudinal cohort study conducted in central and northeastern Massachusetts, USA, that started in 2018. The details about study recruitment and procedures have been published elsewhere [15]. Briefly, individuals were eligible if they were 65 years of age or older, planned to live in the area for at least three years, and were able to walk with or without assistive devices. Individuals were excluded if they were unable to do interviews or questionnaires due to visual or auditory impairments, not living independently, had severe memory issues measured using the Short Portable Mental Status Questionnaire (SPMSQ), were unable to do all study-related activities independently, or did not report their fall status during the study (<5%). Direct mailing was the primary method of recruiting participants. Recruitment presentations were given in group settings such as senior centers, older adults' day care centers, and veterans' organizations. Individuals expressing interest in the study were contacted by research staff who provided details about the study and conducted eligibility screening. A total of 716 community-dwelling adults were enrolled in the study during the period 2018–2023. The study protocol was approved by the University of Massachusetts Lowell Institutional Review Board (#: 20-142-LI-XPD; 21-017-LI-XPD). All study participants provided written informed consent.

### 2.2. Measures

A fall was defined as unintentionally coming to rest on the ground or a lower surface. Information related to falls and associated conditions was obtained from a monthly falling calendar followed by a standardized questionnaire, both administered by trained professional research staff. Participants used the monthly falling calendar to record daily if they had fallen and mailed back the monthly falling calendar to the study office at the end of each month. Research staff then called participants who had reported falls and asked for details about their fall(s) including circumstances, location, footwear worn, potential influence of lighting or medication, and whether they were injured and went to the hospital. An indoor fall was defined as occurring inside any building other than a parking garage, and an outdoor fall was defined as occurring outside any building or in a parking garage. The number of indoor and outdoor falls was collected from June 12, 2018, to December 31, 2023.

Recreational walking was defined as walking for exercise for at least 10 min in the participants' neighborhood, not including walking to stores or businesses. Participants were asked about their frequency of recreational walking habits in the past month, with the following distribution: 20.67% did not walk at all, 7.54% walked less than once a month, 10.89% walked 1–3 times a month, 11.59% walked 1–2 times per week, 18.16% walked 3–4 times per week, 12.29% walked 5–6 times per week, and 18.85% walked at least once a day. The information about recreational walking was collected at participants' baseline visits. For analysis, the responses were summarized into two groups, in which 0 corresponded to less than once per week, and 1 corresponded to at least once per week.

### 2.3. Covariables

Sociodemographic variables included participant's age, self-reported gender (women, men, other), self-reported race and ethnicity, geographic region (urban, suburban, rural), household income (<$50K, $50K or more, unknown), and educational attainment (high school or lower, college, beyond college). Physical health variables included self-rated health (good-excellent, poor-fair), body mass index (BMI) (<25 kg/m^2^, 25–29.9 kg/m^2^, ≥30 kg/m^2^), the number of medical comorbidities, and bodily pain (none-mild, moderate-severe). Functional status was assessed by the five-timed chair stand test (<15.96 s, ≥15.96 s), hand grip [male: low (<30 kg), medium (30–36 kg), high (>36 kg); female: low (<20 kg), medium (20–23 kg), high (>23 kg)], activities of daily living (ADL) [16] (no difficulty, at least some difficulty), and instrumental activities of daily living (IADL) [17] (no difficulty, at least some difficulty). Mental health variables included the Center for Epidemiologic Studies Depression Scale (CES-D) [18], the Beck Anxiety Inventory (BAI) [19], the Perceived Stress Scale (PSS-4) [20], and the modified Brief Resilience Scale [21]. Lifestyle behaviors were evaluated using the Physical Activity Scale for the Elderly (PASE) [22], measures of social support and social activity (≤17, >17 times per month) [23], and by determining whether participants drank alcoholic beverages (no, yes), smoked (no, yes), or lived alone (no, yes). The level of concern about falling was measured by the Falls Efficacy Scale International (FES-I) [24].

### 2.4. Statistical analyses

Participant characteristics were summarized overall and stratified by gender. Continuous variables were described using means and standard deviations (*Mean* ± *SD*); their differences by gender were compared using *t*-tests or Wilcoxon rank-sum tests. Categorical variables were described using frequency and percentages [*n* (%)], and gender differences were compared using Chi-squared or Fisher exact tests.

Mixed effects negative binomial models were performed to estimate gender differences in associations between recreational walking and rates of outdoor and indoor falls, separately for men and women. Unadjusted negative binomial models were used to estimate the crude relationships between recreational walking and indoor/outdoor falls (Model 1). Model 2 included all covariables to estimate the adjusted associations between recreational walking and indoor/outdoor falls. An interaction term between female sex and recreational walking was added into Model 2 to estimate gender differences in the associations (Model 3). For a parsimonious model, we included age and race/ethnicity as a priori variables, and other covariables with statistically significant associations with indoor/outdoor fall rates were retained. Using a stepwise elimination approach, statistically insignificant covariables were eliminated sequentially. Collinearity was assessed using variance inflation factor (VIF) and generalized VIF, with a threshold of VIF/GVIF > 10 indicating a collinearity issue. The GVIF was performed using the car package in R version 4.1.1.

The percentages of missing values for covariates ranged from 0% to 1.7% (1.7% for comorbidity variables and less than 1% for other covariates). Participants with complete data were included in models at each level of adjustment. Model fit was evaluated by the Akaike information criterion (AIC) and the Bayesian information criterion (BIC). Data were analyzed using Stata 18 (Stata Corp., College Station, TX, USA). Two-sided *p*-values < 0.05 were considered statistically significant.

## 3. Results

### 3.1. Participant characteristics

Among the 716 participants, 394 (55.0%) were female and 322 (45.0%) were male; no participants selected the “other” category. Additionally, 464 (64.9%) self-reported as non-Hispanic White, 378 (52.9%) lived in urban areas, 244 (34.1%) in suburban areas, and 93 (13.0%) in rural areas, and the *mean* (*SD*) age of participants at baseline visits was 74.08 (6.29) years old. Compared to men, a higher proportion of women reported lower household income, lower educational attainment, fair or poor health, poorer physical function, and lived alone. Moreover, women had more anxiety symptoms, more medical comorbidities, and greater concern of falling, as well as lower levels of physical activity and resilience (Table 1). About 61% of participants reported they engaged in recreational walking at least once a week. The prevalence of it was 60.4% among women and 61.5% among men, and there were not any significant gender differences in the frequency of recreational walking (*p* = 0.77).

The *mean* (*SD*) follow-up for women was 2.20 (0.08) years, 2.01 (0.08) years for men, and there were no gender differences in follow-up time (*p* = 0.10). A total of 353 participants (49.30%) reported experiencing at least one fall during the study period. Compared to men, women had a significantly lower rate of outdoor falls (32 *vs*. 40 per 100 person-years, *p* = 0.01). Gender differences in the rate of indoor falls were not statistically significant (women *vs*. men: 31 *vs*. 34 per 100 person-years, *p* = 0.34) (Table 1).

### 3.2. Association between recreational walking and indoor falls

Table 2 shows the associations between recreational walking and indoor fall rates. More frequent recreational walking was significantly associated with a lower indoor fall rate (Model 1); the association remained significant after adjusting for covariables (Model 2). Both Models 3 and 4 found that the interaction between female sex and recreational walking was significant, indicating gender differences in the associations between recreational walking and indoor fall rates. In the parsimonious model (Model 4), higher rates of indoor falls were associated with non-Hispanic White race and ethnicity [IRR (95% *CI*): 2.36 (1.72, 3.25)], a higher level of anxiety [1.05 (1.03, 1.07)], and a greater concern about falling [1.03 (1.01, 1.05)]. Obesity was associated with a lower rate of indoor falls 0.70 (0.50, 1.00); being overweight did not show a significant association.

In Model 4, for men, recreational walking was not significantly associated with indoor fall rate [IRR (95% *CI*): 0.90 (0.61, 1.32)]. However, women engaging in recreational walking had a 62% lower indoor fall rate [IRR (95% *CI*): 0.38 (0.21, 0.71)] than those who did not engage. Model 4 had the lowest BIC, indicating the best model performance.

### 3.3. Association between recreational walking and outdoor falls

Recreational walking was not significantly associated with outdoor fall rates in both unadjusted and adjusted models [unadjusted IRR: 1.14 (0.86, 1.51); adjusted IRR: 1.09 (0.81, 1.46)]. No significant interaction between females and recreational walking was found. However, participants who self-reported as non-Hispanic White or had difficulties in ADL were more likely to experience outdoor falls (Table 3).

**Table 1.** Characteristics of participants (overall and by gender).

**Project**	**Total (*N* = 716)**	**Women (*N* = 394)**	**Men (*N* = 322)**	***p*-value for gender diff.**
**Sociodemographic variables**				
**Age at baseline visit, y, *mean* (*SD*)**	74.08 (6.29)	73.91 (6.23)	74.28 (6.37)	0.48
**Race and ethnicity, *n* (%)**				<0.001
**Non-Hispanic White**	464 (64.9)	235 (59.6)	229 (71.3)	
**Others^1^**	251 (35.1)	159 (40.4)	92 (28.7)	
**Education, *n* (%)**				<0.001
**High school or lower**	200 (27.9)	127 (32.2)	73 (22.7)	
**College**	321 (44.8)	181 (45.9)	140 (43.5)	
**Beyond college**	195 (27.2)	86 (21.8)	109 (33.9)	
**Household income, *n* (%)**				<0.001
**<$50K**	278 (38.8)	180 (45.7)	98 (30.4)	
**$50K or more**	340 (47.5)	150 (38.1)	190 (59.0)	
**Unknown**	98 (13.7)	64 (16.2)	34 (10.6)	
**Areas, *n* (%)**				0.12
**Rural**	93 (13.0)	43 (10.9)	50 (15.6)	
**Suburban**	244 (34.1)	132 (33.5)	112 (34.9)	
**Urban**	378 (52.9)	219 (55.6)	159 (49.5)	
**Physical health**				
**BMI, *n* (%)**				0.01
**<25 kg/m^2^**	212 (29.8)	135 (34.6)	77 (24.0)	
**25–29.9 kg/m^2^**	294 (41.4)	144 (36.9)	150 (46.7)	
**≥30 kg/m^2^**	205 (28.8)	111 (28.5)	94 (29.3)	
**# of medical comorbidities, *mean* (*SD*)**	1.83 (1.32)	2.05 (1.39)	1.56 (1.16)	<0.001
**Bodily pain past four weeks, *n* (%)**				0.05
**None-mild**	535 (74.8)	283 (72.0)	252 (78.3)	
**Moderate-severe**	180 (25.2)	110 (28.0)	70 (21.7)	
**Self-rated health, *n* (%)**				<0.001
**Good-excellent**	566 (79.1)	292 (74.1)	274 (85.1)	
**Poor-fair**	150 (20.9)	102 (25.9)	48 (14.9)	
**Functional status**				
**Five-timed chair stand test^2^, *n* (%)**				
**High function**	454 (63.4)	251 (63.7)	203 (63.0)	0.86
**Low function**	262 (36.6)	143 (36.3)	119 (37.0)	
**ADL, *n* (%)**				
**No difficulty**	580 (81.2)	311 (79.1)	269 (83.8)	0.11
**At least some difficulty**	134 (18.8)	82 (20.9)	52 (16.2)	
**IADL, *n* (%)**				
**No difficulty**	417 (58.2)	200 (50.8)	217 (67.4)	<0.001
**At least some difficulty**	299 (41.8)	194 (49.2)	105 (32.6)	
**Hand grip (kg)^3^, *n* (%)**				0.73
**Low**	225 (31.6)	128 (32.7)	97 (30.2)	
**Medium**	240 (33.7)	132 (33.7)	108 (33.6)	
**High**	248 (34.8)	132 (33.7)	116 (36.1)	
**Lifestyle behaviors**				
**Recreational walking, *n* (%)**				0.77
**Less once/week**	280(39.1)	156 (39.6)	124 (38.5)	
**At least once/week**	436(60.9)	238 (60.4)	198 (61.5)	
**Current drinking alcohol, *n* (%)**				<0.001
**No**	277 (38.8)	174 (44.3)	103 (32.1)	
**Yes**	437 (61.2)	219 (55.7)	218 (67.9)	
**Current smoker, *n* (%)**				0.14
**No**	676 (94.5)	377 (95.7)	299 (93.1)	
**Yes**	39 (5.5)	17 (4.3)	22 (6.9)	
**Live alone, *n* (%)**				<0.001
**No**	517 (72.3)	260 (66.0)	257 (80.1)	
**Yes**	198 (27.7)	134 (34.0)	64 (19.9)	
**PASE, *mean* (*SD*)**	1.38 (0.79)	1.30 (0.80)	1.49 (0.77)	<0.001
**Social support, *mean* (*SD*)**	3.00 (0.93)	3.00 (0.89)	3.00 (0.97)	0.59
**Social activity^4^**				0.06
**Fewer**	376 (52.6)	194 (49.4)	182 (56.5)	
**More**	339 (47.4)	199 (50.6)	140 (43.5)	
**Mental health**				
**PSS-4, *mean* (*SD*)**	3.07 (2.73)	3.08 (2.75)	3.06 (2.71)	0.95
**Resilience, *mean* (*SD*)**	3.80 (0.75)	3.77 (0.76)	3.85 (0.74)	0.05
**CES-D, *mean* (*SD*)**	6.71 (6.95)	7.02 (7.26)	6.33 (6.53)	0.40
**BAI, *mean* (*SD*)**	6.18 (7.02)	6.66 (7.01)	5.58 (7.00)	<0.001
**Fall-related variables**				
**FES-I, *mean* (*SD*)**	23.99 (8.48)	25.45 (9.05)	22.21 (7.36)	<0.001
**Indoor fall rate (per 100 person-years)**	32	31	34	0.34
**Outdoor fall rate (per 100 person-years)**	36	32	40	0.01

Note:^1^ Others in this study included 143 (20%) Asian, 76 (10.6%) Hispanic, and 33 (4.6%) unknown. ^2^ The cutoff point for the five-timed chair stand test was selected based on the *mean* value. Low function *vs*. high function was defined as <15.96 s *vs*. ≥15.96 s. ^3^ The cutoff point of hand grip was selected based on its distribution. Low function *vs*. medium function *vs*. high function was defined as <30 kg, 30–36 kg, >36 kg among men; <20 kg, 20–23 kg, >23 kg among women. ^4^ Social activity was measured using the sum of monthly frequency of activities. Fewer activities *vs*. more activities was defined as ≤17 *vs*. >17 times per month.

**Table 2.** Associations between recreational walking and indoor fall rates among older adults.

**Project**	**Indoor fall rate**			
	**Model 1 IRR (95%*CI*) (*N* = 716)**	**Model 2 IRR (95%*CI*) (*N* = 686)**	**Model 3 IRR (95%*CI*) (*N* = 686)**	**Model 4 IRR (95%*CI*) (*N* = 709)**
**Female**		0.89 (0.67, 1.17)	1.17 (0.81, 1.69)	1.21 (0.84, 1.76)
**Recreational walking**	0.60 (0.45,0.79)	0.69 (0.52, 0.90)	0.93 (0.63, 1.37)	0.90 (0.61, 1.32)
**Female × Recreational walking**			0.56 (0.33, 0.95)	0.52 (0.31, 0.87)
**Sociodemographic**				
**Age**		1.01 (0.99, 1.04)	1.01 (0.99, 1.04)	1.01 (0.99, 1.03)
**non-Hispanic White (ref: others)**		2.85 (1.90, 4.26)	2.97 (1.98, 4.45)	2.36 (1.72, 3.25)
**College (ref: high school or less)**		1.33 (0.96, 1.86)	1.30 (0.93, 1.82)	
**Beyond college (ref: high school or lower)**		1.49 (1.00, 2.22)	1.42 (0.95, 2.12)	
**Household income ≥ $50K (ref: <$50K)**		1.05 (0.73, 1.50)	1.03 (0.72, 1.47)	
**Household income unknown (ref: <$50K)**		0.68 (0.44, 1.05)	0.68 (0.44, 1.05)	
**Suburban (ref: rural area)**		0.98 (0.61, 1.58)	0.97 (0.60, 1.56)	
**Urban (ref: rural area)**		1.41 (0.88, 2.25)	1.40 (0.87, 2.23)	
**Physical health**				
**25–29.9 kg/m^2^ (ref: <25 kg/m^2^)**		0.87 (0.63, 1.20)	0.86 (0.62, 1.19)	0.88 (0.64, 1.21)
**≥30 kg/m^2^ (ref: <25 kg/m^2^)**		0.69 (0.48, 1.00)	0.67 (0.46, 0.96)	0.70 (0.50, 1.00)
**# of medical comorbidities**		1.04 (0.92, 1.17)	1.03 (0.91, 1.16)	
**Moderate-severe body pain (ref: none-mild body pain)**		0.94 (0.67, 1.33)	0.96 (0.68, 1.35)	
**Poor-fair health (ref: good-excellent health)**		1.12 (0.71, 1.77)	1.13 (0.72, 1.78)	
**Functional status**				
**Low function in five-timed chair stand test (ref: high function)**		0.95 (0.71, 1.27)	0.95 (0.71, 1.26)	
**At least some difficulty in ADL (ref: no difficulty)**		1.00 (0.69, 1.44)	1.00 (0.69, 1.45)	
**At least some difficulty in IADL (ref: no difficulty)**		0.92 (0.65, 1.30)	0.92 (0.66, 1.30)	
**Medium group in hand grip (ref: low)**		1.15 (0.82, 1.62)	1.20 (0.85, 1.69)	
**High group in hand grip (ref: low)**		1.12 (0.78, 1.61)	1.16 (0.81, 1.67)	
**Lifestyle behaviors**				
**Current drinking alcohol (ref: not drinking)**		0.95 (0.70, 1.31)	0.91 (0.67, 1.25)	
**Current smoker (ref: not smoking)**		1.18 (0.71, 1.94)	1.15 (0.70, 1.89)	
**Living alone (ref: not living alone)**		0.97 (0.71, 1.32)	1.00 (0.73, 1.36)	
**PASE**		0.85 (0.70, 1.04)	0.86 (0.70, 1.05)	
**Social support**		1.01 (0.86, 1.18)	1.01 (0.86, 1.18)	
**More social activities (ref: fewer social activities)**		0.88 (0.67, 1.16)	0.92 (0.70, 1.21)	
**Mental Health**				
**PSS-4**		1.00 (0.94, 1.06)	0.99 (0.94, 1.06)	
**Resilience**		1.23 (1.01, 1.48)	1.20 (0.99, 1.45)	
**CES-D**		1.02 (1.00, 1.05)	1.02 (1.00, 1.05)	
**BAI**		1.05 (1.02, 1.07)	1.05 (1.02, 1.07)	1.05 (1.03, 1.07)
**Fall-related variables**				
**FES-I**		1.03 (1.00, 1.05)	1.02 (1.00, 1.05)	1.03 (1.01, 1.05)
**Model fit**				
**AIC**		2244.52	2241.79	2252.55
**BIC**		2435.81	2438.70	2320.36

Note: Model 1: unadjusted association between recreational walking and indoor falls; Model 2: model 1 + covariables; Model 3: model 2 + interaction between female and recreational walking; Model 4: parsimonious model.

**Table 3.** Associations between recreational walking and outdoor fall rate among older adults.

**Project**	**Outdoor fall rate**		
	**Model 1 IRR (95% *CI*) (*N* = 716)**	**Model 2 IRR (95% *CI*) (*N* = 686)**	**Model 3 IRR (95% *CI*) (*N* = 686)**
**Female**		0.72 (0.54, 0.97)	0.64 (0.41, 1.00)
**Recreational walking**	1.14 (0.86, 1.51)	1.09 (0.81, 1.46)	0.99 (0.66, 1.47)
**Female × Recreational walking**			1.23 (0.70, 2.15)
**Sociodemographic**			
**Age**		0.98 (0.96, 1.01)	0.98 (0.96, 1.01)
**Non-Hispanic White (ref: others)**		2.90 (1.89, 4.45)	2.86 (1.86, 4.39)
**College (ref: high school or less)**		0.92 (0.64, 1.31)	0.93 (0.65, 1.33)
**Beyond college (ref: high school or less)**		1.17 (0.78, 1.75)	1.19 (0.79, 1.79)
**Household income ≥$50K (ref: <$50K)**		0.97 (0.66, 1.43)	0.98 (0.66, 1.44)
**Household income unknown (ref: <$50K)**		1.00 (0.66, 1.51)	1.00 (0.66, 1.51)
**Suburban (ref: rural area)**		0.86 (0.57, 1.32)	0.86 (0.57, 1.32)
**Urban (ref: rural area)**		0.97 (0.63, 1.50)	0.97 (0.63, 1.50)
**Physical health**			
**25–29.9 kg/m^2^ (ref: <25 kg/m^2^)**		0.64 (0.46, 0.91)	0.65 (0.46, 0.91)
**≥30 kg/m^2^ (ref: <25 kg/m^2^)**		0.46 (0.31, 0.69)	0.47 (0.32, 0.70)
**# of medical comorbidities**		1.01 (0.89, 1.15)	1.01 (0.89, 1.16)
**Moderate-severe body pain (ref: none-mild body pain)**		1.42 (0.96, 2.09)	1.42 (0.96, 2.09)
**Poor-fair health (ref: good-excellent health)**		0.98 (0.56, 1.72)	0.97 (0.55, 1.71)
**Functional status**			
**Low function in five-timed chair stand test (ref: high function)**		0.88 (0.64, 1.20)	0.88 (0.64, 1.20)
**At least some difficulty in ADL (ref: no difficulty)**		1.54 (1.01, 2.36)	1.54 (1.00, 2.36)
**At least some difficulty in IADL (ref: no difficulty)**		0.75 (0.51, 1.10)	0.75 (0.51, 1.10)
**Medium group in hand grip (ref: low)**		1.24 (0.84, 1.81)	1.22 (0.83, 1.80)
**High group in hand grip (ref: low)**		1.23 (0.83, 1.82)	1.22 (0.82, 1.80)
**Lifestyle behaviors**			
**Current drinking alcohol (ref: not drinking)**		1.16 (0.83, 1.63)	1.17 (0.83, 1.64)
**Current smoker (ref: not smoking)**		1.33 (0.80, 2.24)	1.34 (0.80, 2.25)
**Living alone (ref: not living alone)**		0.78 (0.55, 1.11)	0.78 (0.55, 1.11)
**PASE**		1.13 (0.92, 1.38)	1.12 (0.92, 1.37)
**Social support**		1.07 (0.90, 1.29)	1.07 (0.90, 1.29)
**More social activities (ref: fewer social activities)**		0.92 (0.70, 1.22)	0.91 (0.69, 1.21)
**Mental Health**			
**PSS-4**		1.01 (0.94, 1.09)	1.01 (0.94, 1.09)
**Resilience**		0.93 (0.76, 1.14)	0.93 (0.76, 1.14)
**CES-D**		0.98 (0.96, 1.01)	0.98 (0.96, 1.01)
**BAI**		1.03 (0.99, 1.06)	1.03 (0.99, 1.06)
**Fall-related variables**			
**FES-I**		1.01 (0.99, 1.04)	1.01 (0.99, 1.04)
**Model fit**			
**AIC**		2335.21	2336.70
**BIC**		2526.49	2533.62

Note: Model 1: unadjusted association between recreational walking and outdoor fall rate; Model 2: model 1 + covariables; Model 3: model 2 + interaction between female and recreational walking.

## 4. Discussion

This prospective cohort study provides novel information about gender differences in the relationships between recreational walking and rates of indoor and outdoor falls among community-dwelling older women and men. Key findings from the study included the following: a) a higher frequency of recreational walking was associated with a lower rate of indoor falls, but showed no effect on outdoor falls; b) women had a significant association between recreational walking and indoor fall rate that was not observed in men; c) non-Hispanic White race and ethnicity, fear of falling, and anxiety symptoms were associated with higher rates of indoor falls.

### 4.1. Recreational walking and lower indoor falls

Previous studies have found that mental health issues, poor physical function, and a higher burden of chronic diseases such as cardiovascular disease are risk factors for indoor falls [8],[25]. The effects of walking on these risk factors have been reported. Recreational walking has been shown to be associated with reduced mental health issues such as anxiety, depression, and stress [26]–[28]. Additionally, a study found that higher intensity of recreational walking was associated with better mental health [29]. The mechanism of the effects of recreational walking on mental health could involve a reduction in amygdala activity. The amygdala, responsible for processing emotional stimuli, becomes overactive under adverse conditions, such as re-exposure to traumatic reminders [30],[31]. Overactivity increases the risk of mental health issues such as anxiety, depression, and stress [31]–[33]. Research indicates that recreational walking can reduce amygdala activity, thereby improving mental health [34]. In terms of physical function and chronic diseases, systematic reviews have found that walking can reduce the risk factors of cardiovascular disease, such as lowering blood pressure and increasing aerobic capacity [35]. Furthermore, walking can improve physical function including increasing lower-body strength as well as static and dynamic balance [36],[37]. The positive effects on mental health, physical function, and cardiovascular diseases could provide potential explanations for the observed association between recreational walking and indoor fall rates.

Consistent with previous studies, the current study did not find a significant association between recreational walking and outdoor fall rates [38]. This lack of association may be attributed to a complex interplay between the physical benefits of recreational walking and the impacts of environmental hazards on outdoor falls. While recreational walking is associated with better functional abilities and physical health, which could reduce rates of outdoor falls, these protective effects may not sufficiently counteract the impacts of environmental hazards on outdoor falls. Research has found that about 73% of outdoor falls were precipitated by environmental factors such as uneven or wet surfaces, tripping, or slipping on objects [8]. Therefore, even though older adults engaging in recreational walking have protective factors against outdoor falls, their exposure to environmental risks may counteract these benefits, contributing to the observed lack of significant association.

### 4.2. Recreational walking and lower indoor falls in women (but not men)

The observed gender difference in the relationship between recreational walking and indoor fall rate is another important finding, which could be explained by gender differences in walking companionship. Compared to men, women were more likely to do physical activity with their friends or participate in group walking [39],[40], which serves as both physical and social activities. Group walking not only increases physical activity but also provides psychological benefits that improve mental well-being [41]–[43]. These benefits include distracting from negative feelings and increased release of neurotransmitters including endorphins, dopamine, and serotonin, which are known to contribute to mental health [44]–[47].

Furthermore, improved physical activity and mental health have been found to enhance cognitive function and functional ability and reduce fear of falling, all of which were associated with lower rates of indoor falls [25],[48]–[51]. Therefore, women's greater likelihood to engage in group walking may offer combined physical and mental benefits that men are less likely to experience, explaining the observed reduction in indoor fall rates among women.

### 4.3. Major risk factors with higher indoor fall rate

Non-Hispanic White ethnicity was associated with higher rates of indoor and outdoor falls, but the results were different from the Maintenance of Balance, Independent Living, Intellect, and Zest in the Elderly of Boston study (MOBILIZE Boston Study), which suggested no difference in indoor fall rates among non-Hispanic White older adults but an increased rate of outdoor falls [25]. This difference may stem from methodological differences, as the MOBILIZE Boston Study primarily focused on urban areas, whereas our HANS study included urban, suburban, and rural areas. Our HANS study found that the proportion of non-Hispanic White participants living in highly car-dependent suburban and rural areas was higher than that of the other groups, which potentially might have led to spending more time indoors, being less physically active, and having higher risks of indoor falls. Additionally, our models adjusted for covariables, including sociodemographic factors, amount of physical activity, functional status, and mental health. Moreover, our study showed that higher levels of fear of falling were associated with higher indoor fall rates, consistent with findings from prior studies [9]. Importantly, a meta-analysis found a positive association between anxiety and falls, but our specific findings suggested that anxiety symptoms may be related more to indoor rather than outdoor falls [52].

### 4.4. Strengths and limitations

This study has several strengths. First, it contributes to the understanding of gender differences in recreational walking and falls among community-dwelling older adults. Further, we found that gender differences should be considered in fall prevention; this study may be the first to provide estimates of differences between women and men in terms of recreational walking and its effect on indoor and outdoor falls. There are also some limitations in this study. First, recall bias or age-related memory issues could affect the reliability and accuracy of the collected data. To minimize this issue, monthly falling calendars and phone interviews shortly after a fall was reported were used to improve accuracy. Second, the measurement of recreational walking is based on frequency but lacks duration and intensity, which could also affect fall risks. Recreational walking was only collected at the baseline visit, and participants' recreational walking habits may change over the study period; this could affect the associations between recreational walking and falls. Finally, in addition to the risk factors considered in the current study, specific medical conditions, medication use, and environmental factors such as living space, public safety, or flat terrain could affect the associations. Further studies are needed to account for these factors.

## 5. Conclusions

In conclusion, the primary finding of this study was that for older women, but not men, a higher frequency of recreational walking was associated with lower rates of subsequent indoor falls. These results provide specific information about gender differences in the relationship between recreational walking and indoor and outdoor fall rates and elucidate the social and health factors associated with indoor and outdoor falls. These findings provide new insights and hypotheses about how recreational walking may affect falls differently in men and women.

## Use of AI tools declaration

The authors declare they have not used Artificial Intelligence (AI) tools in the creation of this article.
